# Ex vivo anti-microbial efficacy of various formaldehyde releasers against antibiotic resistant and antibiotic sensitive microorganisms involved in infectious keratitis

**DOI:** 10.1186/s12886-020-1306-8

**Published:** 2020-01-15

**Authors:** Daeryl E. Amponin, Joanna Przybek-Skrzypecka, Mariya Zyablitskaya, Anna Takaoka, Leejee H. Suh, Takayuki Nagasaki, Stephen L. Trokel, David C. Paik

**Affiliations:** 1 Department of Ophthalmology, Edward S. Harkness Eye Institute, Columbia University College of Physicians and Surgeons, 635 West 165th Street, Research Annex Room 715, New York, NY 10032 USA; 2 Department of Experimental and Clinical Pharmacology, Medical University of Warsaw, Warsaw, Poland; 3 Department of Ophthalmology, Medical University of Warsaw, Warsaw, Poland

**Keywords:** Sodium hydroxymethylglycinate, Tissue cross-linking, Infectious keratitis, Methicillin-resistant *Staphylococcus aureus* (MRSA), Antibiotic resistant microorganisms, Formaldehyde releasers (FARs)

## Abstract

**Background:**

Corneal infections with antibiotic-resistant microorganisms are an increasingly difficult management challenge and chemically or photochemically cross-linking the cornea for therapy presents a unique approach to managing such infections since both direct microbial pathogens killing and matrix stabilization can occur simultaneously. The present study was undertaken in order to compare the anti-microbial efficacy, in vitro, of 5 candidate cross-linking solutions against 5 different microbial pathogens with relevance to infectious keratitis.

**Methods:**

In vitro bactericidal efficacy studies were carried out using 5 different FARs [diazolidinyl urea (DAU), 1,3-bis(hydroxymethyl)-5,5-dimethylimidazolidine-2,4-dione (DMDM), sodium hydroxymethylglycinate (SMG), 2-(hydroxymethyl)-2-nitro-1,3-propanediol (NT = nitrotriol), 2-nitro-1-propanol (NP)] against 5 different microbial pathogens including two antibiotic-resistant species [methicillin-sensitive *Staphylococcus aureus (MSSA),* methicillin-resistant *Staphylococcus aureus (MRSA),* vancomycin-resistant *Enterococcus (VRE), Pseudomonas aeruginosa (PA),* and *Candida albicans (CA)].* Standard in vitro antimicrobial testing methods were used.

**Results:**

The results for MSSA were similar to those for MRSA. DAU, DMDM, and SMG all showed effectiveness with greater effects generally observed with longer incubation times and higher concentrations. Against MRSA, 40 mM SMG at 120 min showed a > 95% kill rate, *p* < 0.02. Against VRE, 40 mM DAU for 120 min showed a > 94% kill rate, *p* < 0.001. All FARs showed bactericidal effect against *Pseudomonas aeruginosa*, making PA the most susceptible of the strains tested. *Candida* showed relative resistance to these compounds, requiring high concentrations (100 mM) to achieve kill rates greater than 50%.

**Conclusion:**

Our results show that each FAR compound has different effects against different cultures. Our antimicrobial armamentarium could potentially be broadened by DAU, DMDM, SMG and other FARs for antibiotic-resistant keratitis. Further testing in live animal models are indicated.

## Background

Corneal scars, remnants of infectious keratitis, are one of the leading causes of blindness and visual impairment worldwide [1] and a continuous rise in the incidence of bacterial and fungal keratitis has been reported recently [2]. The US Centers for Disease Control and Prevention estimates that over 2 million people are infected with drug-resistant microbes annually in the US [3, 4]. This includes a soaring number of multi-resistant microorganisms affecting the human cornea (i.e. *Pseudomonas aeruginosa (PA),* methicillin- resistant *Staphylococcus aureus (MRSA)* and methicillin- susceptible *Staphylococcus aureus (MSSA*)) [5, 6]. Rising rates of resistance to first and second-line traditional antibiotic treatment, such as the fluoroquinolone ciprofloxacin, has been observed [7]. Thus, the number of blind individuals as a result of corneal infections will rise as our ability to effectively treat infectious keratitis diminishes secondary to the increasing development of microbial pathogen resistance. This underlines a need to seek alternatives to available antibiotic treatment protocols [8].

Several strategies to combat multi-drug resistance are under development. Some of the approaches include: the development of new classes of antibiotics (i.e. teixobactin, which shows activity against *Staphylococcus aureus* and *Mycobacterium tuberculosis*) [9], novel application of well-known antibiotic (i.e. chloramphenicol for fungal infection [10]), synergistic combinations of existing antibiotics [11], systemic antibiotics [12], as well as potentiator molecules (especially for gram negative bacteria) that serve to increase bacterial membrane permeability [13]. One of the potentially new approaches to multi-drug resistant keratitis treatment is riboflavin-UVA photochemical corneal cross-linking (or CXL). CXL was originally developed for the treatment of keratoconus [14]. Covalent modification of fibrillar collagens and the extracellular matrix molecules results in tissue strengthening and can halt ectatic progression [15] . A growing body of evidence shows the benefits of PACK-CXL, a trademark for application of the CXL techniques to infectious keratitis [16].

Importantly, PACK-CXL has been shown to have equal or improved bactericidal efficacy against antibiotic resistant strains of *Pseudomonas, Enterococcus,* and *Staphylococcus aureus* [17]. Furthermore, an overwhelming number of reports have shown that CXL is effective as an adjunct to standard antibiotic agents [18, 19] for bacterial keratitis. CXL has also been used with success as a primary therapy for infectious keratitis due to bacterial causes [20, 21]. That being said, it is important to note that CXL is contraindicated for the treatment of herpetic keratitis [22] and can cause reactivation of latent herpes [23]. CXL also appears to be less effective against fungal infections, where the PACK-CXL clinical literature is less convincing [24]. Other drawbacks to CXL include the potential UVA exposure risks and issues surrounding corneal epithelial debridement. For these reasons, we are investigating the use of topical therapeutic cross-linking solutions to provide a new cross-linking option for patients.

Formaldehyde releasers (FARs) are a group of over 60 chemicals widely used in the textile and cosmetics industries [25]. They differ from one another in terms of toxicity, water solubility, molecular weight, hydrophobicity, mutagenicity and bioavailability [25]. We are developing them for clinical ophthalmic use in the form of a cross-linking solution. This could provide a new option for corneal tissue stabilization in keratoconus and post –LASIK ectasias. Initial studies had focused on the nitroalcohols, a subgroup of FARs [26]. The aim of the present study was to assess the antimicrobial efficacy and identify differences between 5 selected formaldehyde-releasing agents: diazolidinyl urea (DAU), 1,3-bis(hydroxymethyl)-5,5-dimethylimidazolidine-2,4-dione (DMDM), sodium hydroxymethylglycinate (SMG), 2-(hydroxymethyl)-2-nitro-1,3-propanediol (NT = nitrotriol), and 2-nitro-1-propanol (NP) [see Table 1] against 5 different keratitis pathogens. The present study serves as an extension of our previous work using SMG only [33]. Considering the interesting results from that study, we sought to look for other FARs with potential application as topical cross-linking agents for infectious keratitis.

**Table 1:** Formaldehyde-releasing agents (FARs) included in the study, chemical names, acronyms, molecular weights and structure

Chemical name	Acronym	Moles of FA released per 1 mol of FAR	Molecular weight	Predicted octanol/water partition coefficient, LogP	Toxicity (LD50, dermal, rabbit)	Structure
Diazolidinyl urea (1-[3,4-bis(hydroxymethyl)-2,5-dioxoimidazolidin-4-yl]-1,3-bis(hydroxymethyl)urea)	DAU	4	278.22	−5.395 ± 0.866 [27]	> 2000 mg/kg [28]	
1,3-Bis(Hydroxymethyl)-5,5-dimethylimidazolidine-2,4-dione	DMDM	2	188.18	−2.3 [29]	> 2000 mg/kg [30]	
Sodium hydroxymethylglycinate	SMG	1	127.07	−1.197 [31]	> 2000 mg/kg [32]	
2-(hydroxymethyl)-2-nitro-1,3-propanediol	NT	3	151.12	−0.115 ± 0.77 [27]	NA	
2-nitro-1-propanol	NP	1	105.09	−0.066 ± 0.269 [27]	NA	
Formaldehyde	FA		30.03	0.350 ± 0.145 [27]	NA

**Table 2:** Summary of the experimental conditions

Bacteria strain	FAR’s concentration, mM	Time of incubation, min	Microorganism concentration, CFUs
Methicillin-sensitive *Staphylococcus aureus*	0, 20, 40, 100	60, 120	10^4^, 10^4/2^
Methicillin-resistant *Staphylococcus aureus*	0, 20, 40, 100	60, 120	10^4^, 10^4/2^
Vancomycin-resistant *Enterococcus*	0, 20, 40, 100	60, 120	10^4^, 10^4/2^
*Pseudomonas aeruginosa*	0, 20, 40, 100	60, 120	10^4^, 10^4/2^
*Candida albicans*	0, 20, 40, 100	60, 120	10^4^, 10^4/2^

## Methods

### Chemicals

The bactericidal effect of five different formaldehyde-releasing agents were studied. Key chemicals were as follows: a) sodium hydroxymethylglycinate 50% (SMG) [Suttocide™, Ashland, Columbus OH, USA], b) 2-nitro-1-propanol (NP), c) diazolidinyl urea (DAU) [Sigma Aldrich, Saint Louis, USA], d) 1,3-bis(hydroxymethyl)-5,5-dimethyl-2,4-imidazolidinedione (DMDM) and e) 2-(hydroxymethyl)-2-nitro-1,3-propanediol (NT) [Chemistry Connection LLC, Conway AR, USA]. BBL™ Trypticase™ Soy Broth, BBL™ Trypticase™ Soy Agar, Difco™ Sabouraud Dextrose Broth, Difco D/E Neutralizing Broth [Fisher Scientific, Waltham, MA, USA] were used for bacteria growth. Adult bovine serum albumin (BSA) was bought from Sigma-Aldrich Corp. (St. Louis, MO, USA). All FARs dilutions were made with balanced salt solution, BSS Plus® [Alcon Laboratory Inc., Forth Worth, TX, USA] and were prepared within 60 min of the experiments apart from NT and NP which were prepared 24 h before the experiment due to their prolonged formaldehyde release.

### Bacteria strains

The following microorganisms were obtained from the American Type Culture Collection (ATCC, Manassas, VA, USA): methicillin-resistant *Staphylococcus aureus* [(MRSA) ATCC 33592], *Staphylococcus aureus* (ATCC 6538), *Pseudomonas aeruginosa* (ATCC 27853), *Candida albicans* (ATTC 11651). VRE was a clinical isolate #10988 from Columbia University Medical Center, Department of Surgery.

### Experimental procedure

Treatment conditions (FAR concentration, incubation period and use of NB) are summarized in Table 2. Pathogens were grown from a slant in either a Trypticase Soy Broth (TSB) with 10% Albumin for MRSA, MSSA, PA, and VRE or a Difco Sabouraud Dextrose Broth (DB) with 10% BSA for CA. The optical density of each pathogen was determined using a spectrophotometer set at 600 nm and zeroed using respective broths containing 10% BSA protein. From the exponential growth phase, 50 μL of 5 × 10^3^ to 10^5^ colony forming units (CFU) per mL were added to a 96 well flat bottom assay plate. Each well was treated with a final concentration of 20 mM, 40 mM, 100 mM of FAR by pipetting 50 μL of FAR dissolved in a balanced salt solution (BSS) into each well. Control wells were treated with 50 μL of BSS. Following addition of FAR, the lid was placed on the assay plate, and it was gently rocked back and forth 5 times to mix the FAR and pathogen. After a 60–120 min treatment period, 200 μL of Difco D/E Neutralizing Broth (39 mg/mL) was pipetted into each well to neutralize the SMG, and mixed by pipetting up and down five times. The resulting mixture of the pathogen, FAR, and neutralizing agent was pipetted onto an BBL trypticase soy agar plate and evenly spread with an L-spreader. Plates were incubated upside down in a Forma-Steri-Cycle CO_2_ incubator for 20–28 h, except Candida which was incubated for a longer time period (48–58 h) due to its slower growth rate. Plates were then manually counted by the naked eye and recorded. During the counting process, each colony was marked on the bottom of the culture plate with a fine point marking pen in order to assure accurate counting.

### Statistical analysis

Each final concentration of FAR (0 [control], 20 mM, 40 mM, 100 mM) was tested on 3 to 6 plates for every pathogen and every incubation time (30 min, 1 h, 2 h). The kill rate for each plate was calculated by comparing the number of colonies on the plate and the mean value of the colonies on the control plates. Two-way ANOVA was used to compare the differences in the means of the kill rate, FARs concentration, and the time of the treatment. As for the graphs, the mean kill rate for every plate for each FAR dosage were plotted and a linear regression model was generated. Wilcoxon rank sum test was applied to compare two groups. For all of the analyses *p* < 0.05 was considered statistically significant. The data and models were analyzed using the software STATA 13.1 (College Station, TX, USA). These computational statistical analyses were carried out with the assistance the Columbia University biostatistical core service.

## Results

In this study, we tested the anti-microbial efficacy of five FARs against 5 different relevant pathogens and a main point of this study has been to delineate potential differences in efficacy between the different compounds. The effect of FARs differed among microorganisms and compounds and their concentrations itself. SMG proved to be the most effective overall. Compared to the control group the most prominent bactericidal effect for 60 min incubations were obtained for 100 mM SMG against MRSA (kill rate 96%, SD 5%) and PA (kill rate 96%, SD 3%). This is particularly important as these two species are particularly prevalent antibiotic resistant organisms. For VRE the best effect was obtained with DMDM 100 mM at 60 min (kill rate 91%, SD 7%). As would be expected based on the relationship between contact time and kill rate, using 120 min incubation time, the kill rate exceeded 90% in 11 different means (DAU 100 mM and SMG 100 mM for all bacteria tested: MRSA, MSSA, PA, VRE; DAU 40 mM for VRE; SMG 40 mM for MRSA and DMDM 100 mM for PA) (Table 3). That being said, greater killing effects were not always observed by extending the exposure time from 60 min to 120 min for a given concentration. That is, when comparing kill rates between 60 min and 120 min at the same concentration. The reasons for this inconsistency is unclear, however, explanations include the possibility of polymerization effects occurring as a result of released free formaldehyde (i.e. formaldehyde polymerizing with itself) as well as possible reactions with the FAR products resulting in formation of either the starting material or additional reaction products. An example of this was reported previously by our group [26].

**Table 3:** 60- and 120-min incubation time experiment: Mean Kill Rates ±SD of different compounds on five different bacterial strains and *p* values (p* compares 0 and the relevant dose of the same FAR for 60 min, p** compares 0 and the relevant dose of each FAR for 120 min)

Bacteria strain	Compound	Concentration (mM)	60 min		120 min	
			Mean Kill Rate ± SD (%)	p*	Mean Kill Rate ± SD (%)	p**
MSSA	DAU	20	64 ± 13	0.000	38 ± 15	0.239
		40	55 ± 36	0.000	80 ± 10	0.017
		100	92 ± 6	0.000	93 ± 5	0.006
	DMDM	20	−2 ± 77	0.92	55 ± 22	0.091
		40	48 ± 13	0.01	49 ± 30	0.132
		100	52 ± 48	0.006	88 ± 11	0.010
	SMG	20	35 ± 16	0.055	81 ± 9	0.016
		40	49 ± 27	0.009	87 ± 10	0.010
		100	88 ± 2	0.000	100 ± 4	0.004
	NT	20	−20 ± 14	0.262	−48 ± 31	0.137
		40	57 ± 36	0.003	−47 ± 108	0.150
		100	52 ± 47	0.006	−60 ± 115	0.067
	NP	20	39 ± 9	0.037	−66 ± 47	0.044
		40	38 ± 46	0.042	−61 ± 123	0.062
		100	39 ± 24	0.035	18 ± 13	0.565
MRSA	DAU	20	27 ± 29	0.042	59 ± 25	0.119
		40	51 ± 12	0.000	94 ± 4	0.016
		100	86 ± 16	0.000	97 ± 1	0.013
	DMDM	20	−15 ± 60	0.223	75 ± 4	0.052
		40	44 ± 38	0.001	85 ± 2	0.029
		100	80 ± 12	0.000	88 ± 3	0.023
	SMG	20	9 ± 12	0.582	83 ± 6	0.032
		40	34 ± 8	0.111	95 ± 4	0.015
		100	96 ± 5	0.000	100 ± 1	0.011
	NT	20	20 ± 4	0.241	69 ± 17	0.073
		40	51 ± 9	0.017	2 ± 57	0.964
		100	62 ± 1	0.005	55 ± 45	0.144
	NP	20	52 ± 7	0.003	−5 ± 35	0.892
		40	44 ± 24	0.038	−59 ± 57	0.123
		100	60 ± 36	0.001	122 ± 224	0.002
VRE	DAU	20	50 ± 16	0.000	68 ± 25	0.000
		40	64 ± 29	0.000	94 ± 2	0.000
		100	87 ± 11	0.000	98 ± 3	0.000
	DMDM	20	−1 ± 100	0.925	25 ± 15	0.000
		40	62 ± 15	0.000	44 ± 2	0.000
		100	91 ± 7	0.000	85 ± 2	0.000
	SMG	20	70 ± 7	0.001	61 ± 2	0.000
		40	73 ± 7	0.000	84 ± 2	0.000
		100	87 ± 9	0.000	93 ± 2	0.000
	NT	20	17 ± 6	0.404	−5 ± 12	0.456
		40	−4 ± 15	0.856	7 ± 2	0.29
		100	−2 ± 15	0.902	18 ± 5	0.009
	NP	20	−4 ± 7	0.798	65 ± 16	0.000
		40	−5 ± 2	0.750	34 ± 15	0.000
		100	−11 ± 12	0.517	48 ± 31	0.000
PA	DAU	20	51 ± 11	0.000	19 ± 18	0.113
		40	67 ± 10	0.000	70 ± 26	0.000
		100	86 ± 9	0.000	99 ± 1	0.000
	DMDM	20	64 ± 33	0.000	76 ± 17	0.000
		40	69 ± 10	0.000	16 ± 37	0.000
		100	79 ± 12	0.000	97 ± 2	0.000
	SMG	20	61 ± 3	0.000	74 ± 10	0.000
		40	85 ± 2	0.000	89 ± 9	0.000
		100	96 ± 3	0.000	99 ± 1	0.000
	NT	20	55 ± 15	0.000	7 ± 15	0.496
		40	83 ± 3	0.000	19 ± 9	0.112
		100	71 ± 3	0.000	24 ± 28	0.017
	NP	20	70 ± 7	0.000	52 ± 6	0.000
		40	81 ± 11	0.000	78 ± 5	0.000
		100	83 ± 5	0.000	86 ± 17	0.000
CA	DAU	20	24 ± 4	0.804	5 ± 10	0.290
		40	20 ± 24	0.846	7 ± 8	0.163
		100	72 ± 10	0.013	53 ± 8	0.000
	DMDM	20	6 ± 35	0.585	23 ± 10	0.000
		40	42 ± 14	0.001	37 ± 10	0.000
		100	59 ± 15	0.000	60 ± 11	0.000
	SMG	20	3 ± 25	0.274	0 ± 3	0.887
		40	33 ± 21	0.626	2 ± 7	0.627
		100	38 ± 30	0.430	4 ± 4	0.493
	NT	20	16 ± 11	0.690	3 ± 18	0.525
		40	53 ± 12	0.109	1 ± 5	0.790
		100	42 ± 3	0.375	54 ± 4	0.000
	NP	20	42 ± 20	0.333	0 ± 8	0.948
		40	25 ± 2	0.934	10 ± 5	0.049
		100	63 ± 13	0.039	86 ± 4	0.000

In general, the results with the nitroalcohols (NA) were not satisfactory. The two NAs tested, NT and NP demonstrated limited potential against MSSA, MRSA and VRE although both showed some effectiveness against PA at 60 min incubation. NAs tend to release free FA slowly by comparison with other FARs (unpublished data). This may account for the lack of antibacterial effectiveness shown by the NAs. Surprisingly, the NAs did fairly well against CA, an organism that proved to be troublesome for the other FARs tested. In summary, the FAR/pathogen pairings that showed the most consistent trends (that is, both dose and time dependency) were as follows: SMG against MSSA, MRSA, and PA; DAU against MRSA and VRE; DMDM against MRSA.

A description of results based on the organism tested is now included:

### *Staphylococcus aureus* (methicillin sensitive = MSSA and methicillin resistant = MRSA) (Fig. 1a and b and Table 3)

The results for MRSA and MSSA were similar for all the compounds. In other words, the effectiveness of a given FAR was similar against either MSSA or MRSA and this is to be expected, given that they are both Staphylococcus species. DAU, DMDM, and SMG all showed some effectiveness with greater effects observed with the longer incubation time of 120 min. There was also a significant concentration dependency for these three agents with higher concentrations having greater efficacy than lower.

**Fig. 1:**
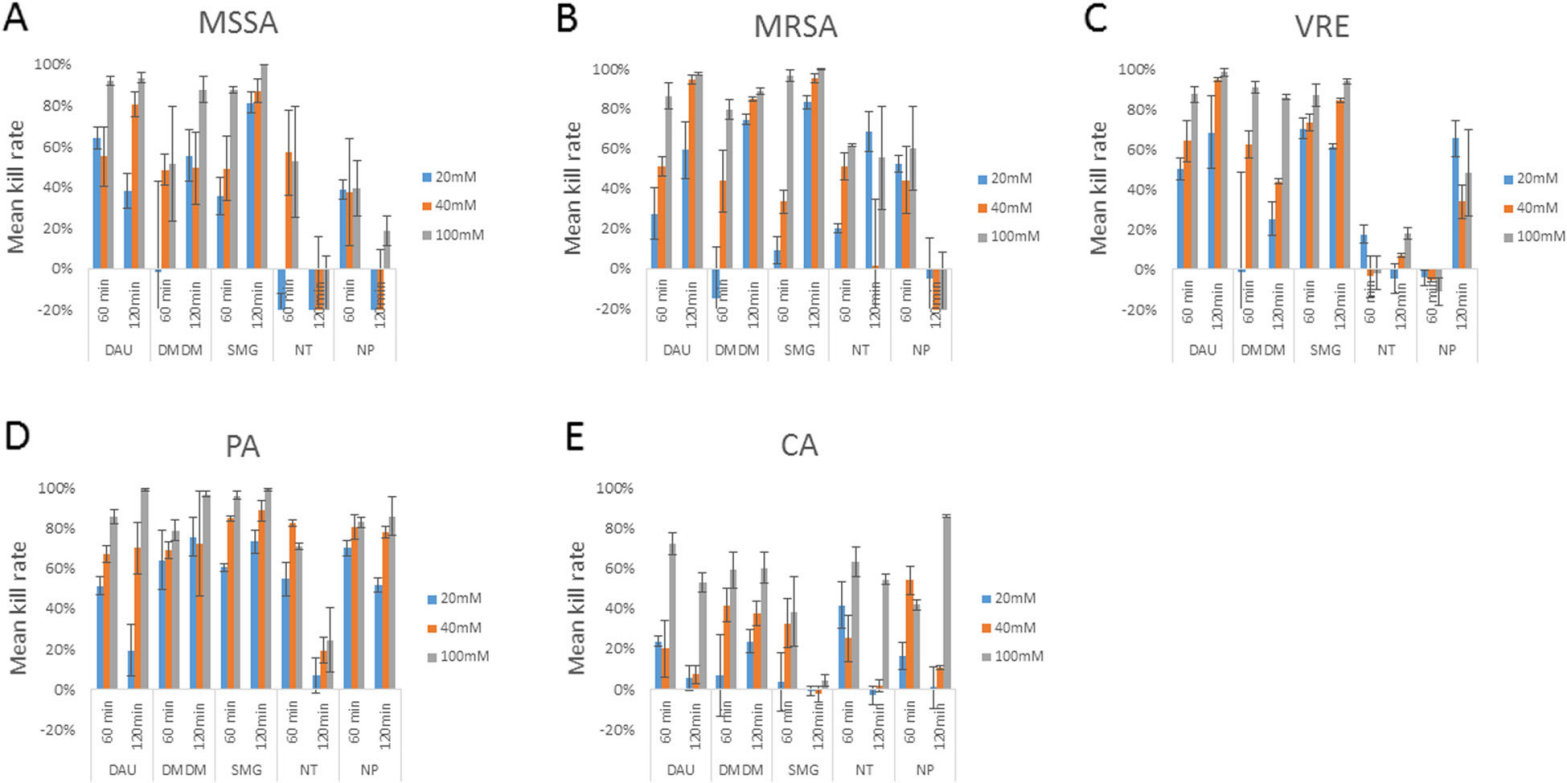
**A.** Bactericidal effect of DAU, DMDM, SMG, NT and NP against MSSA tested in three concentrations (20 mM, 40 mM and 100 mM). Results expressed as a kill rate compared to the control group (1-(mean colonies count in control group/mean colonies count in tested group) *100[%]), 60- and 120-min incubation of the compound with bacteria solution (time of incubation in brackets). **B.** Bactericidal effect of DAU, DMDM, SMG, NT and NP against MRSA tested in three concentrations (20 mM, 40 mM and 100 mM). Results expressed as a kill rate compared to the control group (1-(mean colonies count in control group/mean colonies count in tested group) *100[%]), 60 min incubation of the compound with bacteria solution. **C.** Bactericidal effect of DAU, DMDM, SMG, NT and NP against VRE tested in three concentrations of each FAR (20 mM, 40 mM and 100 mM). Results expressed as a kill rate compared to the control group (1-(mean colonies count in control group/mean colonies count in tested group) *100[%]), 60- and 120-min incubation of the compound with bacteria solution (seeding density 10^4^/2). **D.** Bactericidal effect of DAU, DMDM, SMG, NT and NP against Pseudomonas aeruginosa tested in three concentrations (20 mM, 40 mM and 100 mM). Results expressed as a kill rate compared to the control group (1-(mean colonies count in control group/mean colonies count in tested group) *100[%]), 60 min incubation of the compound with bacteria solution. **E.** Bactericidal effect of DAU, DMDM, SMG, NT and NP against Candida albicans (seeding density 10^4^/2) tested in three concentrations (20 mM, 40 mM and 100 mM). Results expressed as a kill rate compared to the control group (1-(mean colonies count in control group/mean colonies count in tested group) *100[%]), the comparison of 60- and 120-min incubation of the compound with bacteria solution

MSSA growth was inhibited in a dose-dependent pattern. The results obtained for DAU showed robust kill rate, mean colony count for 100 mM was 14 (SD ± 4.4; *p* < 0001 compared to control group), followed by mean 72 for 40 mM concentration (SD ± 27.6; *p* < 0.001 compared to control group) and 120 for 20 mM concentration (SD ± 50.2; *p* < 0.001 compared to control group). The kill rate for each compound and its concentration, as well as the statistical significance of their differences are summarized in Table 3.

Similar to MSSA, MRSA growth was inhibited in a dose-dependent manner using DAU, SMG, DMDM and NT in 60 min incubation time and for DAU, DMDM and SMG for 120 min incubation time. The mean kill rate for the 100 mM dose was: 1) SMG at 96% (SD ± 5% *p* < 0.0001 compared to the controls), 2) DAU at 86% (SD ± 16%, *p* < 0.0001 versus control) and 3) DMDM at 80% (SD ± 12% *p* < 0.0001 vs controls) for 60 min exposure. Additionally, statistically significant results at *p* < 0.01were obtained for 22 different conditions using all 5 FARs at either 60- or 120-min exposures (Table 3). Figure 1a and b depicts mean kill rates of each compound tested.

### *Enterococcus* (vancomycin resistant = VRE) (Fig. 1c and Table 3)

For VRE, SMG and DAU were the most effective, with DAU showing time and concentration dependency with less time and concentration dependence for SMG, which showed a reasonable kill rate even at the lowest concentration (20 mM) and shortest time (60 min). A statistically significant (*p* < 0.01) bactericidal effect was noted for all concentrations of DAU, DMDM, and SMG against VRE although the effects were greatest with DAU and SMG. That being said, the effect with DMDM at 100 mM for 60 min presented the most robust effect among all conditions (kill rate 91%, SD ± 7, *p* < 0.0001 compared to control group). Contrary to the aforementioned, the NAs NT and NP did not impair bacterial growth at all following a 60 min incubation (Table 3).

### *Pseudomonas aeruginosa* = PA (Fig. 1d and Table 3)

All FARs showed bactericidal effects against PA (*p* < 0.0001 for each compound concentration compared to control group) in 60- and 120-min incubations, making PA the most susceptible of the strains tested to FARs. This is important as this was the only gram-negative rod tested and as such, represents one of an important group of bacteria (i.e. gram-negative rods) with several related species also showing antibiotic resistance, (i.e. being *Klebsiella, E. coli,* and *Enterobacter)*. Furthermore, PA is becoming a major problem pathogen leading to rapid corneal perforation as a result of significant collagenase production. This makes cross-linking therapy with SMG an attractive possibility for preserving tissue, since cross-linking increases the resistance of the tissue collagen to enzymatic digestion. Future studies should examine the effects against specific pseudomonal strains.

SMG was the most effective FAR. The kill rate for SMG 100 mM for a 60 min incubation was 96% (SD ± 3) and 99% (SD ± 1) for 120 min incubations. That being said, at the lowest concentration (20 mM), DMDM exerted stronger inhibition against PA than the equivalent concentration of SMG (20 mM). For the majority of compounds, the results show high effectiveness of each compound at 60- and 120-min incubations, with the longer incubation time resulting in a stronger killing effect. However, there is one FAR (NT) that showed an opposite effect in this regard with decreased killing noted during the longer exposure time. The reason for this is unclear. However, one possibility is the reversibility of the reaction as well as products of the reaction getting involved in secondary reactions [26]. The data is displayed in Fig. 1 and Table 3.

### ***Candida albicans*** **= CA** (Fig. 1e and Table 3)

Finally, CA growth was studied under the same time frame (60, 120 min) as the previous strains of microorganisms. CA showed relative resistance to SMG, a compound with consistently good effects against the bacterial species. Although the average activity of FARs were generally lower for CA than for the bacteria strains, we did observe some trends of potential effects as follows: DAU 100 mM at 60 and 120 min (*p* < 0.0001), DMDM 20 mM for 120 min (*p* < 0.0001), DMDM 40 mM for 60 and 120 min (*p* < 0.0001), DMDM 100 mM for 60 and 120 min (*p* < 0.0001), NT 20 and 100 mM for 60 and 120 min (*p* < 0.0001), NP 100 mM for 120 min (*p* < 0.0001). The highest kill rate was obtained for NP 100 mM for 120 min (kill rate 86%, SD ± 4, *p* < 0.001). In addition, it is interesting to note that the NAs performed better against CA than against the other bacteria tested. Figure 1e and Table 3 show the detailed information acquired in the CA experiments.

## Discussion

The emergence of bacterial resistance to traditional antibiotics has become a serious problem in ophthalmology. The latest reports from India suggest that 80% of MRSA strains are resistant to available antibiotics, while the same is true for 20% of MSSA [34]. PA is known clinically as a rapid mutator that can lead to the development of extended spectrum B-lactamase producing variants (ESBL) [35, 36]. New strategies are actively being sought in order to address this concern. One of them, riboflavin-UVA photochemical corneal cross-linking (CXL), has been studied for over a decade now. This procedure was initially used to induce cross-linking (CXL) to stabilize the cornea in keratoconus but is now actively being used to treat corneal infections (PACK-CXL = photoactivated chromophore for keratitis-CXL). There are drawbacks, however, due primarily to the riboflavin photosensitizer and UVA light requirements (UVA risks include cataract formation and retinal degeneration, and direct keratocyte toxicity). CXL is also less effective in deep fungal corneal infections owing to a therapeutic cross-linking effect that is limited to the anterior stroma [37], and is contraindicated for herpetic keratitis, where exacerbations can occur [38, 39]. Therefore, to address these challenges, we are developing a topical approach using formaldehyde releasing compounds (FARs) with a goal of omitting UVA light exposure. In this study we evaluated the therapeutic effect of 5 FARs on 4 bacterial and 1 fungal strain.

The results indicate that different FARs have different microbicidal effects against the 5 pathogens tested. A summary of the results suggests that DAU, DMDM, and SMG all could be potentially used as Staphylococcus drugs (MRSA and MSSA). DAU and SMG, but less so DMDM, also showed promising effects on VRE and even at lower concentrations (20 mM, 40 mM).

Regarding the mechanism of the interaction between FARs and bacteria growth, it seems likely that the induced chemistry that is responsible for extracellular matrix modification could also cause microbial cytotoxicity. In our case, free FA released locally. FA is reactive and so it is likely that covalent modification of microbial target substrate molecules, including proteins with reactive groups (amines, tyrosine, cysteines, etc.), is central to the microbicidal effect [40].

DAU is an allantoin derivative, where allantoin reacts with four equivalents of formaldehyde under basic conditions to form the parent compound [41]. Thus, it has a theoretical yield of 4 mol of FA per mole of DAU. Dilution encourages the decomposition reaction, overcoming possible steric interference and facilitating the separation of the formaldehyde moiety from the mother compound. The actual FA yield of DAU is less than the expected 4 mol, however. Lehmann et al. demonstrated that DAU exists as a mixture of isomers, with “compound BHU” (1-(3,4-bis-hydroxymethyl-2,5-dioxo-imidazolidin-4-yl)-1,3-bis-hydroxymethyl-urea) as the dominant form (30–40%) [42]. It is hypothesized that the remainder consists of many polymers of allantoin-formaldehyde condensation products. Thus, this complex mixture of compounds could account for the lower than expected FA yield.

DMDM is a hydantoin with a theoretical yield of two FA moieties. For DMDM at concentrations from ~ 3 mM to ~ 1.3 M a more alkaline pH (8.5–9 as compared to pH 6–6.5 and pH 4–4.5) and a lower concentration favored higher levels of free FA, consistent with the release characteristics of DAU, which is also increased at lower concentrations [43]. Once FA is liberated from either of the nitrogen atoms of the five-membered ring, the resulting negative charge on the nitrogen atom is delocalized into the π-system provided by both of the adjacent carbonyl moieties. The formation of intramolecular hydrogen bonds between local DMDM molecules stabilizes any additional negative charge.

SMG has a theoretical yield of one mole of FA per mole of SMG. In spite of this lower ratio when compared to the other FARs of this study, SMG appears to release FA more readily than other FARs with strong tissue cross-linking effects [25] and behaves differently from the other FARs in certain regards. Solutions of SMG in water tend to be highly alkaline (Fig. 1) but can be modulated downward with the addition dilute neutral buffers. The other FARs produce neutral solutions and the FA release can be facilitated by the addition of base [44]. It has a molecular weight of 127.07 g/mol. Its small size facilitates its ability to pass through the epithelial barrier to induce cross-linking. In aqueous solutions, SMG decomposes entirely to formaldehyde and its parent compound, sodium glycinate, which is not considered harmful [45].

FARs do not tend to induce microbial resistance in the same manner as traditional antibiotics albeit resistance can occur. A review of this topic has been previously published [46]. Furthermore, because they are broad-spectrum agents, these agents could have unique efficacy against emerging pathogens such as MRSA, VRE, and extended beta-lactamase (EBL)-resistant strains of *Pseudomonas*. Of note, there is precedent for using broad spectrum anti-septic agents such as these for treating infectious keratitis. Human trials indicate that antiseptic agents can be used topically for the treatment of infected tissue fields [46]. Chlorhexidine has been used for *Acanthameoba*, *Staphylococcus aureus* and *Pseudomonas aeruginosa* keratitis [47], iodine for fungal keratitis [48], and hypochlorous acid has been used for infected wounds [49, 50]. Developing single broad-spectrum agents that could take the place of multi-agent therapy for infectious keratitis could be of great patient benefit and is the driving force behind these studies.

Finally, this study only considers direct microbicidal effects and does not account for the potential effects upon the extracellular matrix that induce a resistance to enzymatic digestion. Thus, it is difficult to predict which agents will be most effective in vivo, since different FARs have different protein cross-linking capabilities, in addition to the direct microbicidal effects. Another study limitations are of our concern: FAR toxicity [25], interaction between FARs and other topical drugs, risk of a scar formation. Once again, we emphasize that this is an in vitro study and the effects and considerations for in vivo use can be very different. That being said, these studies do serve as an initial guide to further development and should prompt the testing of these compounds in live animal studies. The results of such future studies hold the promise of significantly increasing our armamentarium against threatening infections caused by highly resistant micro-organisms.

## Conclusions

Our results show that each FAR compound has different effects against different cultures, including antibiotic resistant strains such as MRSA, VRE, and pseudomonas. Thus, the clinically useful antimicrobial armamentarium could be broadened by the addition of DAU, DMDM, SMG and other FARs. These agents could be particularly helpful for treating antibiotic-resistant tissue infections of the cornea (i.e. infectious keratitis) as well as other types of tissue infections. Further testing in live animal models are indicated, as well as trials of compassionate human use.

## Data Availability

The datasets used and analyzed during the current study are available from the corresponding author on reasonable request.

## Abbreviations

BSA: Bovine Serum Albumin
CA: *Candida albicans*
CFU: colony-formulating units
CXL: UVA-riboflavin mediated photochemical cross-linking (also known as the Dresden protocol)
DAU: Diazolidinyl urea
DMDM: 1,3-Bis(Hydroxymethyl)-5,5-dimethylimidazolidine-2,4-dione
FA: Formaldehyde
FAR(s): Formaldehyde releasing agent(s)
MDR: Multi-drug resistant
MRSA: Methicillin-resistant *Staphylococcus aureus*
MSSA: Methicillin-sensitive *Staphylococcus aureus*
NP: 2-nitro-1-propanol
NT: 2-(hydroxymethyl)-2-nitro-1,3-propanediol = nitrotriol
PA: *Pseudomonas aeruginosa*
SMG: Sodium hydroxymethylglycinate
TXL: Therapeutic tissue cross-linking
UVA: Ultraviolet-A irradiation
VRE: Vancomycin Resistant Enterococcus

## References

[1] Tacconelli E, Magrini N. Global Priority List of Antibiotic-Resistant Bacteria to Guide Research, Discovery, and Development of New Antibiotics: World Health Organization; 2017. Available online: https://www.who.int/medicines/publications/global-priority-list-antibiotic-resistant-bacteria/en/.

[2] Cornea/external disease summary benchmarks for preferred practice pattern guidelines: American Academy of Ophthalmology; 2017. Available online: https://www.aao.org/Assets/7c7cc42a-675c-41ee-ba61-fa113c271c43/636492185326530000/bc-2208-pppsummarybenchmarks-17-cornea-pdf.

[3] Austin A, Lietman T, Rose-Nussbaumer J (2017). Update on the Management of Infectious Keratitis. Ophthalmol.

[4] Eurosurveillance editorial team C (2013). CDC publishes report on antibiotic resistance threats in the United States for the first time. Eurosurveillance.

[5] Teweldemedhin M, Gebreyesus H, Atsbaha AH, Asgedom SW, Saravanan M (2017). Bacterial profile of ocular infections: a systematic review. BMC Ophthalmol.

[6] Peng MY, Cevallos V, McLeod SD, Lietman TM, Rose-Nussbaumer J (2018). Bacterial keratitis: isolated organisms and antibiotic resistance patterns in San Francisco. Cornea.

[7] Shalchi Z, Gurbaxani A, Baker M, Nash J (2011). Antibiotic resistance in microbial keratitis: ten-year experience of corneal scrapes in the United Kingdom. Ophthalmol.

[8] Drug Resistance in Infectious Agents (2013). A Global Threat to Humanity. G-SCIENCE ACADEMIES STATEMENTS.

[9] Parmar A, Lakshminarayanan R, Iyer A (2018). Design and syntheses of highly potent Teixobactin analogues against Staphylococcus aureus, methicillin-resistant Staphylococcus aureus (MRSA), and Vancomycin-resistant enterococci (VRE) in vitro and in vivo. J Med Chem.

[10] Joseph MR, Al-Hakami AM, Assiry MM (2015). In vitro anti-yeast activity of chloramphenicol: a preliminary report. J Mycol Med.

[11] Blanco AR, Nostro A, D'Angelo V, D'Arrigo M, Mazzone MG, Marino A (2017). Efficacy of a fixed combination of tetracycline, chloramphenicol, and Colistimethate sodium for treatment of Candida albicans keratitis. Invest Ophthalmol Vis Sci.

[12] Spierer O, Miller D, O'Brien TP (2018). Comparative activity of antimicrobials against Pseudomonas aeruginosa, Achromobacter xylosoxidans and Stenotrophomonas maltophilia keratitis isolates. Br J Ophthalmol.

[13] Zabawa TP, Pucci MJ, Parr TR, Lister T (2016). Treatment of gram-negative bacterial infections by potentiation of antibiotics. Curr Opin Microbiol.

[14] Coskunseven E, Jankov MR, Hafezi F (2009). Contralateral eye study of corneal collagen cross-linking with riboflavin and UVA irradiation in patients with keratoconus. J Refract Surg.

[15] Hafezi F, Kanellopoulos J, Wiltfang R, Seiler T (2007). Corneal collagen crosslinking with riboflavin and ultraviolet a to treat induced keratectasia after laser in situ keratomileusis. J Cataract Refract Surg.

[16] Hafezi F (2012). Significant visual increase following infectious keratitis after collagen cross-linking. J Refract Surg.

[17] Makdoumi K, Backman A (2016). Photodynamic UVA-riboflavin bacterial elimination in antibiotic-resistant bacteria. Clin Exp Ophthalmol.

[18] Said DG, Elalfy MS, Gatzioufas Z (2014). Collagen cross-linking with Photoactivated riboflavin (PACK-CXL) for the treatment of advanced infectious keratitis with corneal melting. Ophthalmol.

[19] Price MO, Tenkman LR, Schrier A, Fairchild KM, Trokel SL, Price FW (2012). Photoactivated riboflavin treatment of infectious keratitis using collagen cross-linking technology. J Refract Surg.

[20] Makdoumi K, Mortensen J, Sorkhabi O, Malmvall BE, Crafoord S (2012). UVA-riboflavin photochemical therapy of bacterial keratitis: a pilot study. Graefes Arch Clin Exp Ophthalmol.

[21] Kymionis George D., Kouroupaki Anna I., Liakopoulos Dimitrios A., Arandjelovic Ivana R., Tsoulnaras Konstantinos I. (2016). Multiorganism, Drug-Resistant Keratitis Treated by Corneal Crosslinking. European Journal of Ophthalmology.

[22] Chan TC, Lau TW, Lee JW, Wong IY, Jhanji V, Wong RL (2015). Corneal collagen cross-linking for infectious keratitis: an update of clinical studies. Acta Ophthalmol.

[23] Papaioannou L, Miligkos M, Papathanassiou M (2016). Corneal collagen cross-linking for infectious keratitis: a systematic review and meta-analysis. Cornea.

[24] Uddaraju M, Mascarenhas J, Das MR (2015). Corneal cross-linking as an adjuvant therapy in the Management of Recalcitrant Deep Stromal Fungal Keratitis: a randomized trial. Am J Ophthalmol.

[25] Babar N, Kim M, Cao K (2015). Cosmetic preservatives as therapeutic corneal and scleral tissue cross-linking agents. Invest Ophthalmol Vis Sci.

[26] Paik DC, Solomon MR, Wen Q, Turro NJ, Trokel SL (2010). Aliphatic beta-nitroalcohols for therapeutic corneoscleral cross-linking: chemical mechanisms and higher order nitroalcohols. Invest Ophthalmol Vis Sci.

[27] SciFinder. Chemical Abstracts Service.

[28] Sigma-Aldrich. Diazolidinyl urea [Material Safety Data Sheet].

[29] ACME-Hardesty C. DMDM hydantoin [Material safety data sheet].

[30] Isenberg SJ, Apt L, Valenton M, Sharma S, Garg P, Thomas PA, Parmar P, Kaliamurthy J, Reyes JM, Ong D, Christenson PD, Del Signore M, Holland GN. Prospective, Randomized Clinical Trial of Povidone-Iodine 1.25% Solution Versus Topical Antibiotics for Treatment of Bacterial Keratitis. Am J Ophthalmol. 2017;176:244–53. 10.1016/j.ajo.2016.10.004 27984024

[31] Sigma-Aldrich. Sodium hydroxymethylglycinate [Material Safety Data Sheet].

[32] Products IS. Sodium hydroxymethylglycinate [Material Safety Data Sheet].

[33] Rapuano PB, Scanameo AH, Amponin DE (2018). Antimicrobial studies using the therapeutic tissue cross-linking agent, sodium Hydroxymethylglycinate: implication for treating infectious keratitis. Invest Ophthalmol Vis Sci.

[34] Lalitha P, Manoharan G, Karpagam R (2017). Trends in antibiotic resistance in bacterial keratitis isolates from South India. Br J Ophthalmol.

[35] Weldhagen GF, Poirel L, Nordmann P (2003). Ambler class a extended-spectrum beta-lactamases in Pseudomonas aeruginosa: novel developments and clinical impact. Antimicrob Agents Chemother.

[36] Sacha P, Wieczorek P, Hauschild T, Zorawski M, Olszanska D, Tryniszewska E (2008). Metallo-beta-lactamases of Pseudomonas aeruginosa--a novel mechanism resistance to beta-lactam antibiotics. Folia Histochem Cytobiol.

[37] Dias J, Diakonis VF, Kankariya VP, Yoo SH, Ziebarth NM (2013). Anterior and posterior corneal stroma elasticity after corneal collagen crosslinking treatment. Exp Eye Res.

[38] Perna JJ, Mannix ML, Rooney JF, Notkins AL, Straus SE (1987). Reactivation of latent herpes simplex virus infection by ultraviolet light: a human model. J Am Acad Dermatol.

[39] Rooney J. F., Straus S. E., Mannix M. L., Wohlenberg C. R., Banks S., Jagannath S., Brauer J. E., Notkins A. L. (1992). UV Light-Induced Reactivation of Herpes Simplex Virus Type 2 and Prevention by Acyclovir. Journal of Infectious Diseases.

[40] Conaway CC, Whysner J, Verna LK, Williams GM (1996). Formaldehyde mechanistic data and risk assessment: endogenous protection from DNA adduct formation. Pharmacol Ther.

[41] Flyvholm MA (2005). Preservatives in registered chemical products. Contact Dermatitis.

[42] Lehmann SV, Hoeck U, Breinholdt J, Olsen CE, Kreilgaard B (2006). Characterization and chemistry of imidazolidinyl urea and diazolidinyl urea. Contact Dermatitis.

[43] Emeis D, Anker W, Wittern KP (2007). Quantitative 13C NMR spectroscopic studies on the equilibrium of formaldehyde with its releasing cosmetic preservatives. Anal Chem.

[44] Solomon MR, O'Connor NA, Paik DC, Turro NJ (2010). Nitroalcohol induced hydrogel formation in amine-functionalized polymers. J Appl Polym Sci.

[45] The Scientific Committee on Costmetic Products and Non-Food Products Intended for Consumers. Opinion concerning the determination of certain formaldehyde releasers in cosmetic products. SCCNFP 2002. Available online: https://webcache.googleusercontent.com/search?q=cache:3dOLXqYJQvgJ:https://ec.europa.eu/health/archive/ph_risk/committees/sccp/documents/out188_en.pdf+&cd=1&hl=pl&ct=clnk&gl=pl&client=safari.

[46] McDonnell Gerald, Russell A. Denver (1999). Antiseptics and Disinfectants: Activity, Action, and Resistance. Clinical Microbiology Reviews.

[47] Bu Ping, Riske Paul S., Zaya Ninef E., Carey Roberta, Bouchard Charles S. (2007). A Comparison of Topical Chlorhexidine, Ciprofloxacin, and Fortified Tobramycin/Cefazolin in Rabbit Models ofStaphylococcusandPseudomonasKeratitis. Journal of Ocular Pharmacology and Therapeutics.

[48] Isenberg SJ, Apt L, Valenton M (2017). Prospective, randomized clinical trial of Povidone-iodine 1.25% solution versus topical antibiotics for treatment of bacterial keratitis. Am J Ophthalmol.

[49] Robson MC, Payne WG, Ko F, Mentis M, Donati G, Shafii SM, Culverhouse S, Wang L, Khosrovi B, Najafi R, Cooper DM, Bassiri M. Hypochlorous acid as a potential wound care agent: part II Stabilized Hypochlorous Acid: Its Role in Decreasing Tissue Bacterial Bioburden and Overcoming the Inhibition of Infection on Wound Healing. J Burns Wounds. 2007;6:e6. PMC1853324 17492051

[50] Odorcic Silvia, Haas Wolfgang, Gilmore Michael S., Dohlman Claes H. (2015). Fungal Infections After Boston Type 1 Keratoprosthesis Implantation. Cornea.

